# CD24 Overexpression Is Associated with Poor Prognosis in Luminal A and Triple-Negative Breast Cancer

**DOI:** 10.1371/journal.pone.0139112

**Published:** 2015-10-07

**Authors:** Mi Jeong Kwon, Jinil Han, Ji Hyun Seo, Kyoung Song, Hae Min Jeong, Jong-Sun Choi, Yu Jin Kim, Seon-Heui Lee, Yoon-La Choi, Young Kee Shin

**Affiliations:** 1 College of Pharmacy, Kyungpook National University, Daegu, Korea; 2 Research Institute of Pharmaceutical Sciences, College of Pharmacy, Kyungpook National University, Daegu, Korea; 3 Gencurix, Inc., Seoul, Korea; 4 Laboratory of Molecular Pathology and Cancer Genomics, Department of Pharmacy, College of Pharmacy, Seoul National University, Seoul, Korea; 5 ABION Inc, Seoul, Korea; 6 The Center for Anti-cancer Companion Diagnostics, School of Biological Science, Institutes of Entrepreneurial BioConvergence, Seoul National University, Seoul, Korea; 7 Laboratory of Cancer Genomics and Molecular Pathology, Samsung Biomedical Research Institute, Samsung Medical Center, Sungkyunkwan University School of Medicine, Seoul, Korea; 8 Department of Nursing Science, College of Nursing, Gachon University, Incheon, Korea; 9 Department of Pathology and Translational Genomics, Samsung Medical Center, Sungkyunkwan University School of Medicine, Seoul, Korea; 10 Department of Health Sciences and Technology, SAIHST, Sungkyunkwan University, Seoul, Korea; INRS, CANADA

## Abstract

CD24 is associated with unfavourable prognoses in various cancers, but the prevalence of CD24 expression and its influence on clinical outcome in subtypes of breast cancers remain unclear. CD24 expression was analyzed by immunohistochemistry in 747 breast cancer tissues, and DNA methylation and histone modification status in the promoter region of CD24 were assessed using bisulfite sequencing and chromatin immunoprecipitation assay. 213 (28.5%) samples exhibited high CD24 expression in the membrane and/or cytoplasm of breast cancer cells, and CD24 overexpression was significantly correlated with the presence of lymph node metastasis and more advanced pathological stage. Patients with CD24-high tumours had significantly shorter patient survival than those with CD24-low tumours. Importantly, multivariate analysis that included tumour size, lymph node metastasis and chemotherapy demonstrated that high CD24 expression is independently associated with poorer survival in luminal A and triple-negative breast cancer (TNBC) subtypes. Furthermore, CD24 gene expression was associated with histone acetylation independent of DNA methylation, suggesting its epigenetic regulation in breast cancer. Our results suggest that CD24 overexpression is an independent unfavourable prognostic factor in breast cancer, especially for luminal A and TNBC subtypes, and CD24 may be a promising therapeutic target for specific subtypes of breast cancer.

## Introduction

Breast cancer, which is the most common cancer in women worldwide, is a heterogeneous disease that is currently classified into four major molecular subtypes, namely luminal A, luminal B, human epidermal growth factor receptor 2 (HER2), and triple-negative breast cancer (TNBC), based on the expression of hormone receptors and HER2 [1–3]. Because each subtype has a distinct clinical behaviour and response to therapy, development of targeted therapy for each molecular subtype is required for the successful treatment of breast cancer. Generally, patients with the luminal A subtype have a better prognosis, whereas those with the HER2 or TNBC subtype have worse clinical outcomes [2–4].

CD24 is a mucin-like cell surface protein with highly variable glycosylation depending on the cell or tissue type [5]. CD24 expression has been detected in various types of carcinomas, whereas it is rarely expressed in normal tissues [6, 7]. Its overexpression during cancer progression and its prognostic significance have been reported for many types of cancer including breast, colorectal, gastric, lung, ovarian, pancreatic, and prostate cancers, supporting the usefulness of CD24 as a cancer marker for diagnosis and prognosis [6–10]. CD24 was also found to promote tumour cell proliferation [11] and invasion in several types of cancer cells [12]. Additionally, in breast cancer, CD24 was demonstrated to increase the proliferation, motility, and invasiveness of breast cancer cells, in line with its role in promoting tumour growth and metastasis *in vivo* [13].

Importantly, CD24 was recently identified as a cancer stem cell marker in various types of cancer including pancreatic and lung cancers [14]. In particular, a CD44^+^/CD24^−/low^ subpopulation was identified to have tumour-initiating properties in breast cancer [15], and its tumourigenic phenotype was demonstrated to be related to stem cell-like properties [16]. Accordingly, CD24 in combination with CD44 is currently considered as a marker for cancer stem cells in breast cancer. In accordance with this finding, CD24 was revealed to be involved in the regulation of stemness and the epithelial to mesenchymal transition in breast cancer cells [17]. However, the association of CD44^+^/CD24^−/low^ population with clinical outcome of patients with breast cancer is unclear.

Previous studies reported that positive CD24 expression is an unfavourable prognostic factor in breast cancer. In the study by Kristiansen *et al*., in 201 breast cancer tissues, CD24 expression was an independent prognostic factor for disease-free survival (DFS) [18]. Surowiak *et al*. in 104 patients with breast cancer revealed that cytoplasmic-membranous CD24 expression is significantly associated with shorter overall survival (OS) and progression-free survival (PFS) in a multivariate analysis [19]. Another study in 643 patients with invasive breast cancer reported that CD24 expression was negatively related to OS only in the hormone receptor-positive group [20]. However, most studies have not assessed the association of CD24 expression with patient outcome according to molecular subtypes of breast cancer and the prognostic significance of CD24 expression in each subtype of breast cancer remains unclear.

Although the clinical significance and function of CD24 in various cancers including breast cancer have been frequently reported, little is known concerning the regulatory mechanism of CD24. CD24 mRNA expression is downregulated by oestrogen in breast cancer cells [21]. The transcript level of CD24 in breast cancer stem cells was observed to be downregulated by Twist [22]. Because the promoter sequence of CD24 was recently confirmed, epigenetic analysis of the promoter region of CD24 has not been performed. However, in terms of epigenetic mechanism, a recent study indicated that the promoter region of CD24, one of the cancer stem cell genes, is unmethylated in breast cancer cells regardless of mRNA levels, whereas the expression of other cancer stem cell genes including CD44, CD133, and Musashi–1 has an inverse correlation with DNA methylation, indicating that other epigenetic mechanisms are involved in the transcriptional regulation of CD24 [23]. However, little is known concerning the epigenetic mechanism of CD24.

In the present study, we assessed the prognostic significance of CD24 expression in subgroups of breast cancer including the molecular subtypes to identify patients who could benefit from a therapy targeting CD24. Epigenetic analysis of the promoter region of CD24 was also performed to elucidate the regulatory mechanism of CD24 expression.

## Materials and Methods

### Ethics Statement

This study was approved by the institutional review board (IRB) of the Samsung Medical Center (Seoul, Korea) and performed in accordance with the Declaration of Helsinki. The study was retrospective and informed consents from the patients involved in the study were not required as per the guidelines of IRB. Patient information was anonymized and de-identified prior to analysis.

### Study population and samples

Our study adhered to the Reporting Recommendations for Tumor Marker Prognostic Studies (REMARK) criteria in the design, analysis, and interpretation of the results [24]. Formalin-fixed, paraffin-embedded specimens were obtained from patients with breast cancer who underwent curative resection for primary tumours with lymph node (LN) dissection at the Samsung Medical Center between January 1995 and December 2002. Patients with stage I–III cancer were included, and they had undergone either mastectomy or breast-conserving surgery followed by radiotherapy, chemotherapy, or hormone therapy, either alone or in combination, according to conventional regimens. Each patient’s medical records were reviewed for clinical information including demographics, extent of disease, type of treatment, and treatment outcome. Pathological parameters included tumour size, LN metastasis, pathological stage, hormone receptor status, p53 status, nuclear grade, and histologic grade. The stage of breast cancer was classified according to the American Joint Committee on Cancer TNM criteria (6^th^ edition). A total of 851 patients with primary invasive breast cancer were included in this study.

Each case was categorised into one of four subtypes according to the expression of hormone receptors including oestrogen receptor (ER) and progesterone receptor (PR), HER2, and basal markers (either epidermal growth factor receptor [EGFR] or cytokeratin 5/6 [CK5/6]) as described previously [25]: 1) luminal A (ER+ or PR+/HER2−), 2) luminal B (ER+ or PR+/HER2+), 3) HER2 (ER−/PR−/HER2+) and 4) TNBC (ER−PR−/HER2−). TNBC was further divided into basal-like breast cancer (ER−/PR−/HER2−/EGFR+ or CK5/6+) and quintuple-negative breast cancer (ER−/PR−/HER2−/EGFR−/CK5/6−).

### Immunohistochemical analysis

For immunohistochemical analysis of CD24, tissue microarray slides were prepared as described previously [25] and they were incubated with monoclonal antibodies against CD24 (1:200; clone SN3b, NeoMarkers, Fremont, CA, USA) for 1 h at room temperature. After washing, the tissue sections were reacted with a biotinylated anti-mouse secondary antibody, followed by incubation with streptavidin-horseradish-peroxidase complex. Slides were washed, and the chromogen was developed for 5 min with liquid 3,3′-diaminobenzidine (Dako, Carpineteria, CA, USA). The immunohistochemical staining was scored independently by two pathologists (YLC and JSC) based on both the intensity of staining and percent of stained tumour cells. The membrane and cytoplasmic staining was counted as positive. The staining intensity was subclassified as follows: 0, negative; 1, weak; 2, moderate; or 3, strong. The positive cells were quantified as a percentage of the total number of tumour cells, and the percentage and the staining intensity were then multiplied in order to generate the immunoreactive score (IS) for each of the tumour specimens. Cases with IS more than 100 were considered as high expression.

### Cell lines and drug treatment

Breast cancer cell lines (HCC1419, SK-BR3, MDA-MB–231, and MDA-MB–435) were maintained in RPMI1640 medium supplemented with 10% foetal bovine serum and antibiotics. MCF–7 cells were maintained in Dulbecco’s modified Eagle’s medium. Breast cancer cell lines were obtained from the American Type Culture Collection or Korean Cell Line Bank. Cells were seeded 1 day before epigenetic drug treatment. Seeded cells were treated with 5 μM 5-aza–2′-deoxycytidine (5-aza-dC; Sigma, St. Louis, MO, USA) for 72 h or 200 nM trichostatin A (TSA; Sigma) for 24 h.

### Flow cytometry

Immunostaining and flow cytometry analyses were performed according to standard procedures. Breast cancer cells were harvested via exposure to 0.05% trypsin/EDTA (Hyclone, South Logan, UT, USA) at 37°C for 3 min and resuspended in fluorescence-activated cell sorting (FACS) buffer with 0.5% bovine serum albumin and 0.1% azide at a concentration of 2×105/100 μl. One hundred microliters of each cell solution were incubated with 5 μl of anti-CD24 antibody (clone SWA11) and 0.5 μl of the isotype controls (eBioscience, San Diego, CA, USA) for 30 min at 4°C in ice water. After washing with PBS, cells were resuspended in RAM-FITC (Abcam, Cambridge, MA, USA) diluted 100-fold with FACS buffer and incubated for 30 min at 4°C in ice water. Samples were analysed by FACSCalibur flow cytometer (BD Biosciences, San Jose, CA, USA).

### Quantitative reverse transcription-polymerase chain reaction (qRT-PCR) and western blotting

Total RNA was isolated from cells using Allprep kit (Qiagen) and reverse-transcribed into cDNA using Superscript™ II First-Strand Synthesis System (Invitrogen) according to the manufacturer’s protocol. Following cDNA synthesis, qRT-PCR was conducted in Light Cycler (Roche Applied Science, Mannheim, Germany) using the primers and probes listed in S1 Table. *PAPOLA* was used as reference gene to normalise gene expression.

Whole cell lysates were extracted using RIPA buffer, and 20 μg of whole cell lysates were used for immunoblotting using primary antibody against CD24 (clone SN3, MS-1278-PABX, NeoMarkers) according to the standard procedures.

### Bisulfite sequencing

Genomic DNA was extracted from breast cancer cell lines using DNeasy genomic DNA extraction kit (Qiagen) and bisulfite-modified using EpiTect Bisulfite kit (Qiagen). CpG islands in the CD24 promoter region and bisulfite sequencing PCR (BSP) primers were predicted using Methyl Primer Express^®^ Software (Life Technologies). The CD24 promoter region sequence for BSP primer design was from GeneBank genomic sequence (GenBank accession no. JN036721). The bisulfite-modified DNA was amplified by BSP primers provided in S1 Table, the PCR product was cloned and subsequently sequenced.

### Chromatin immunoprecipitation (ChIP) assay

ChIP assays were performed using the ChromaFlash™ One-Step ChIP Kit (Epigentek, Farmingdale, NY, USA) according to the manufacturer’s instructions with antibodies against various histone marks used in our previous study [26]. ChIP primers are provided in S1 Table.

### Statistical analysis

OS was defined as the time from the date of surgery for the primary tumour to the date of death or the last follow-up. DFS was calculated from the date of surgery for the primary tumour to the date of recurrence, including loco-regional recurrence and distant metastasis, or death from any cause. Distant metastasis-free survival (DMFS) was defined as the time from the date of surgery for the primary tumour to the date of distant metastasis. Differences in the frequencies of the basic characteristics, clinical parameters, and subtypes were statistically analysed using the chi-squared test. Survival curves were constructed using the Kaplan-Meier method, and the log-rank test was used to compare the mean survival rates across the groups. For the multivariate analysis, Cox regression models were constructed to estimate the adjusted hazard ratios (HRs) of the groups according to clinical variables and CD24 expression. *P* values less than 0.05 were considered statistically significant. All of the statistical analyses were performed using R (http://r-project.org).

## Results

### Correlation between CD24 expression and clinicopathological variables

Cases with unsatisfactory CD24 immunostaining or no invasive cancer component were excluded from 851 cases and a total of 747 patients with informative immunohistochemical results were included in this analysis. The median age of the study population at the time of diagnosis was 47.16 years (range, 23.84–81.21). The majority of cases were infiltrating duct carcinoma (90.5%), and more than half (53.5%) of the cases were LN metastasis-negative. The details on the distribution of clinicopathological factors in the study cohort are listed in Table 1.

**Table 1:** Basic characteristics of the patients and their correlation with CD24 expression.

			All		CD24-high		CD24-low		*P* value
			n = 747		n = 213 (28.5%)		n = 534 (71.5%)		
			No.	(%)	No.	(%)	No.	(%)	
Age		Median (range)	47.16 (23.84–81.21)		48.08 (24.08–80.48)		46.80 (23.84–81.21)		
									0.139
		<50	447	59.8%	118	26.4%	329	73.6%	
		≥50	300	40.2%	95	31.7%	205	68.3%	
Tumour size									0.056
		0–2	300	40.2%	71	23.7%	229	76.3%	
		2.1–5	396	53.0%	127	32.1%	269	67.9%	
		≥5.1	51	6.8%	15	29.4%	36	70.6%	
pT									0.059
		1	298	39.9%	71	23.8%	227	76.2%	
		2	396	53.0%	127	32.1%	269	67.9%	
		3–4	53	7.1%	15	28.3%	38	71.7%	
LN metastasis									**<0.001**
		Negative	400	53.5%	93	23.3%	307	76.8%	
		Positive	347	46.5%	120	34.6%	227	65.4%	
pN									**0.006**
		0	400	53.5%	93	23.3%	307	76.8%	
		1	184	24.6%	62	33.7%	122	66.3%	
		2	84	11.2%	32	38.1%	52	61.9%	
		3	79	10.6%	26	32.9%	53	67.1%	
Pathologic stage									**0.020**
		I	194	26.0%	42	21.6%	152	78.4%	
		II	377	50.5%	110	29.2%	267	70.8%	
		III	176	23.6%	61	34.7%	115	65.3%	
Histologic grade									0.187
		1	83	11.1%	17	20.5%	66	79.5%	
		2	269	36.0%	83	30.9%	186	69.1%	
		3	341	45.6%	97	28.4%	244	71.6%	
		Unknown	54	7.2%	16	29.6%	38	70.4%	
Nuclear grade									0.642
		1	72	9.6%	17	23.6%	55	76.4%	
		2	365	48.9%	106	29.0%	259	71.0%	
		3	284	38.0%	81	28.5%	203	71.5%	
		Unknown	26	3.5%	9	34.6%	17	65.4%	
Chemotherapy									0.337
		No	93	12.4%	31	33.3%	62	66.7%	
		Yes	652	87.3%	182	27.9%	470	72.1%	
		Unknown	2	0.3%	0	0.0%	2	100.0%	
Radiotherapy									0.601
		No	295	39.5%	88	29.8%	207	70.2%	
		Yes	450	60.2%	125	27.8%	325	72.2%	
		Unknown	2	0.3%	0	0.0%	2	100.0%	
Hormone therapy									0.197
	No		285	38.2%	73	25.6%	212	74.4%	
	Yes		452	60.5%	137	30.3%	315	69.7%	
	Unknown		10	1.3%	3	30.0%	7	70.0%	
EIC									0.332
	Negative		365	48.9%	102	27.9%	263	72.1%	
	Positive		162	21.7%	38	23.5%	124	76.5%	
	Unknown		220	29.5%	73	33.2%	147	66.8%	
p53									0.598
	Negative		292	39.1%	83	28.4%	209	71.6%	
	Positive		343	45.9%	90	26.2%	253	73.8%	
	Unknown		112	15.0%	40	35.7%	72	64.3%	
ER									0.638
	Negative		300	40.2%	89	29.7%	211	70.3%	
	Positive		446	59.7%	124	27.8%	322	72.2%	
	Unknown		1	0.1%	0	0.0%	1	100.0%	
PR									0.305
	Negative		414	55.4%	125	30.2%	289	69.8%	
	Positive		332	44.4%	88	26.5%	244	73.5%	
	Unknown		1	0.1%	0	0.0%	1	100.0%	
HER2									**0.047**
	Negative		545	73.0%	144	26.4%	401	73.6%	
	Positive		202	27.0%	69	34.2%	133	65.8%	
EGFR									0.940
	Negative		690	92.4%	196	28.4%	494	71.6%	
	Positive		57	7.6%	17	29.8%	40	70.2%	
CK5/6									0.318
	Negative		688	92.1%	200	29.1%	488	70.9%	
	Positive		59	7.9%	13	22.0%	46	78.0%	
Subtype (4 subtypes)									**0.041**
	Luminal A		362	48.5%	102	28.2%	260	71.8%	
	Luminal B		101	13.5%	30	29.7%	71	70.3%	
	HER2		100	13.4%	39	39.0%	61	61.0%	
	TNBC		183	24.5%	42	23.0%	141	77.0%	
	Unknown		1	0.1%	0	0.0%	1	100.0%	
Subtype (5 subtypes)									0.067
	Luminal A		362	48.5%	102	28.2%	260	71.8%	
	Luminal B		101	13.5%	30	29.7%	71	70.3%	
	HER2		100	13.4%	39	39.0%	61	61.0%	
	Basal		66	8.8%	13	19.7%	53	80.3%	
	QNBC		117	15.7%	29	24.8%	88	75.2%	
	Unknown		1	0.1%	0	0.0%	1	100.0%	

Abbreviations: LN, lymph node; EIC, extensive intraductal component; ER, oestrogen receptor; PR, progesterone receptor; HER2, human epidermal growth factor receptor 2; EGFR, epidermal growth factor receptor; CK5/6, cytokeratin 5/6; TNBC, triple-negative breast cancer, QNBC, quintuple-negative breast cancer. *P* values less than 0.05 are marked in bold.

**Fig 1:**
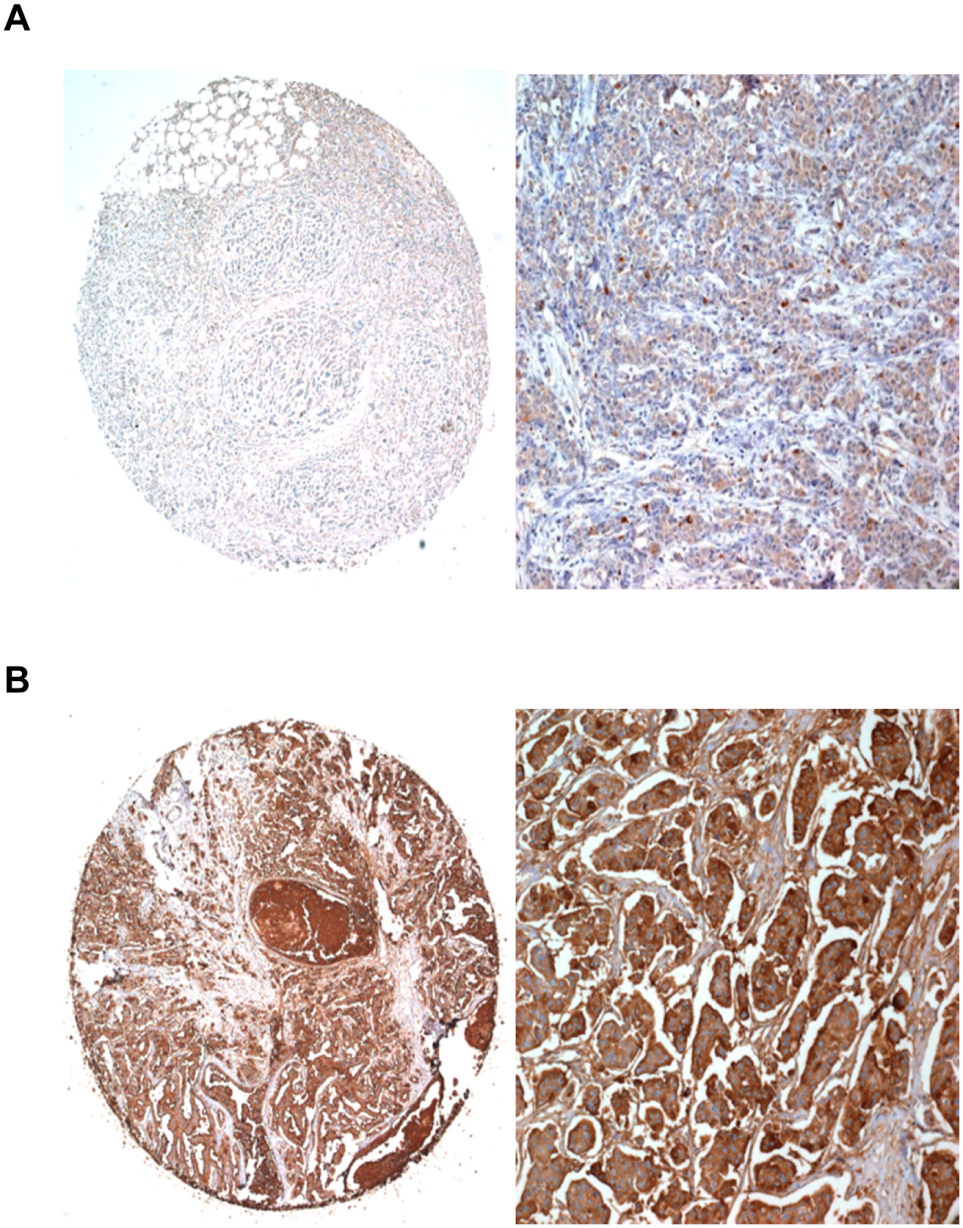
Representative cases of CD24 immunostaining in breast cancer tissue microarray. (A) CD24-low immunoreactivity showing focal weak cytoplasmic staining and (B) CD24-high immunoreactivity showing strong and diffuse in the cytoplasm and membrane of the tumour cells (magnification, ×40 and ×200, respectively).

CD24 immunostaining was predominantly localised in the membrane and/or cytoplasm of breast cancer cells. Of the 747 patients studied, 213 (28.5%) had high CD24 expression, whereas 534 (71.5%) showed low CD24 expression. The immunohistochemical staining of representative cases of low and high CD24 expression is shown in Fig 1. Next, we assessed the relationship of CD24 expression with clinicopathological factors. CD24 expression was significantly correlated with the presence of LN metastasis (*P* < 0.001), higher pN type (regional lymph nodes) (*P* = 0.006) and more advanced pathological stage (*P* = 0.020), suggesting that high CD24 expression is associated with more aggressive breast cancer (Table 1). CD24 expression was more frequently found in HER2-positive breast cancer (34.2%) than in HER2-negative breast cancer (26.4%) (*P* = 0.047). Concerning the expression of CD24 in the four molecular subtypes determined on the basis of immunohistochemical staining for ER, PR, and HER2, CD24 expression varied according to breast cancer subtype (*P* = 0.041); the HER2 subtype had the highest proportion of CD24-high tumours (39.0%). We did not find any significant association of CD24 expression with histologic grade, nuclear grade, p53 status, and ER or PR status in these tumours.

### Association between CD24 expression and clinical outcome in subtypes of breast cancer

The median follow-up times for DFS, DMFS, and OS were 112.0, 115.3, and 122.9 months, respectively. The follow-up times for DFS and DMFS ranged from 0.6 to 232.9 months, and those for OS ranged from 0.5 to 232.9 months. During the follow-up period, 28.4% (212 of 747) of the women had local recurrence and/or distant metastasis, and 26.4% (197 of 747) of the patients died. The 5-year DFS, OS, and DMFS were 77.1, 87.0, and 79.2%, respectively, whereas the 15-year survival rates were 67.3% for DFS, 65.7% for OS, and 71.2% for DMFS.

**Fig 2:**
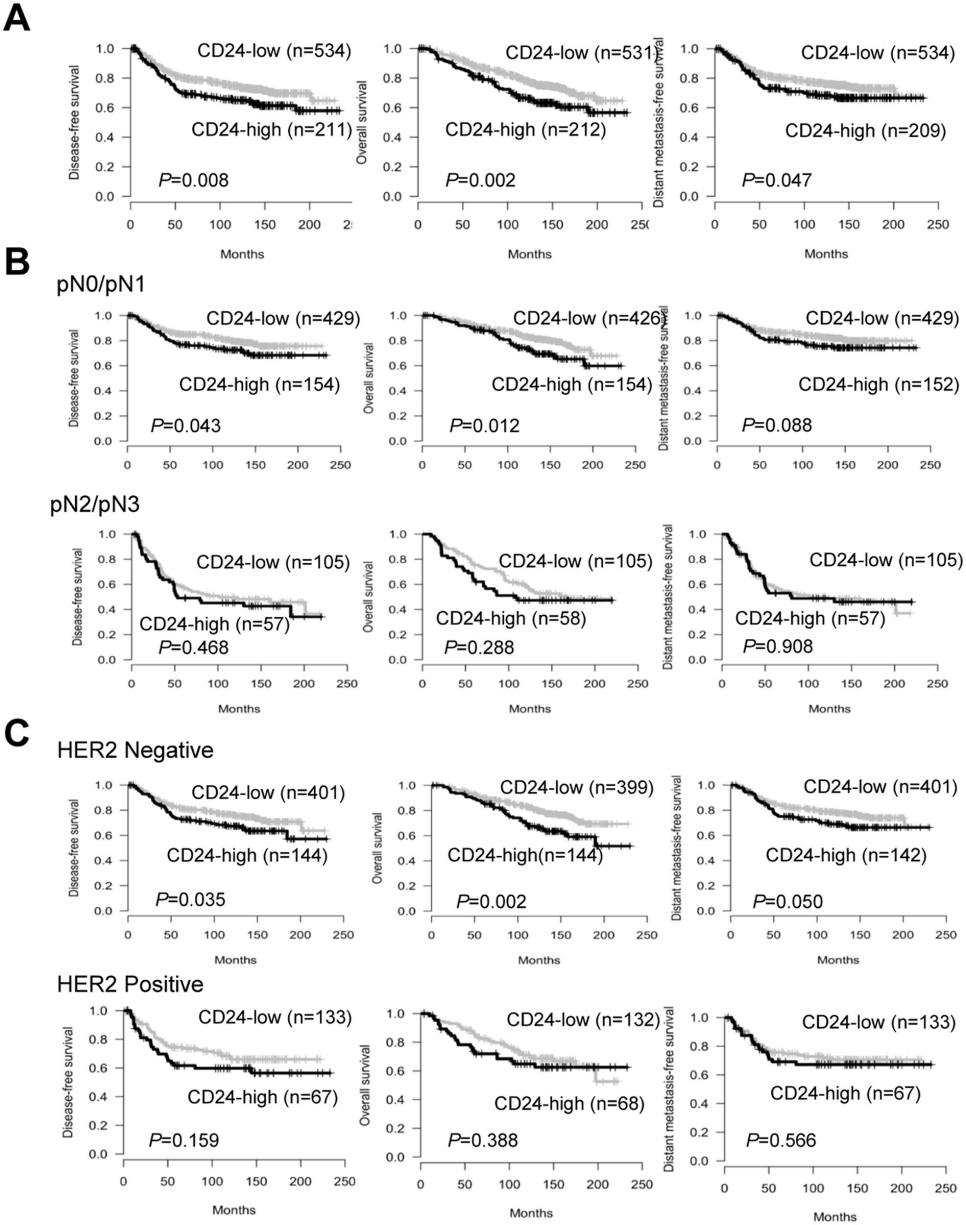
Survival analysis in breast cancer patients based on CD24 expression. (A) Kaplan-Meier curves for disease-free survival (DFS), overall survival (OS), and distant metastasis-free survival (DMFS) according to CD24 expression in all patients. (B) Subgroup survival analysis according to the status of lymph node metastasis, pN0/N1 (top) and pN2/N3 groups (bottom) and (C) according to human epidermal growth factor receptor 2 (HER2) status, HER2-negative (top) and HER2-positive groups (bottom).

To investigate the association of CD24 expression with clinical outcome, patients were divided into CD24-high and CD24-low groups, and survival analyses were performed using the Kaplan-Meier method. As a whole, patients with high CD24 expression had substantially poorer DFS (*P* = 0.008), OS (*P* = 0.002) and DMFS (*P* = 0.047) than those with low CD24 expression (Fig 2A). However, when subgroup analysis was performed according to LN status, high CD24 expression was associated with significantly shorter DFS (*P* = 0.043) and OS (*P* = 0.012) in early-stage breast cancer for pN0/N1 tumours but not pN2/N3 tumours (Fig 2B). Moreover, the significant influence of CD24 expression on OS (*P* = 0.026) was only found in patients with stage I breast cancer (S1 Fig). These results demonstrated that high CD24 expression has an unfavourable impact on DFS and OS in patients with early-stage breast cancer. Subgroup analysis based on HER2 status illustrated that patients with high CD24 expression have significantly worse survival in the HER2-negative subgroup but not in the HER2-positive subgroup (Fig 2C).

**Fig 3:**
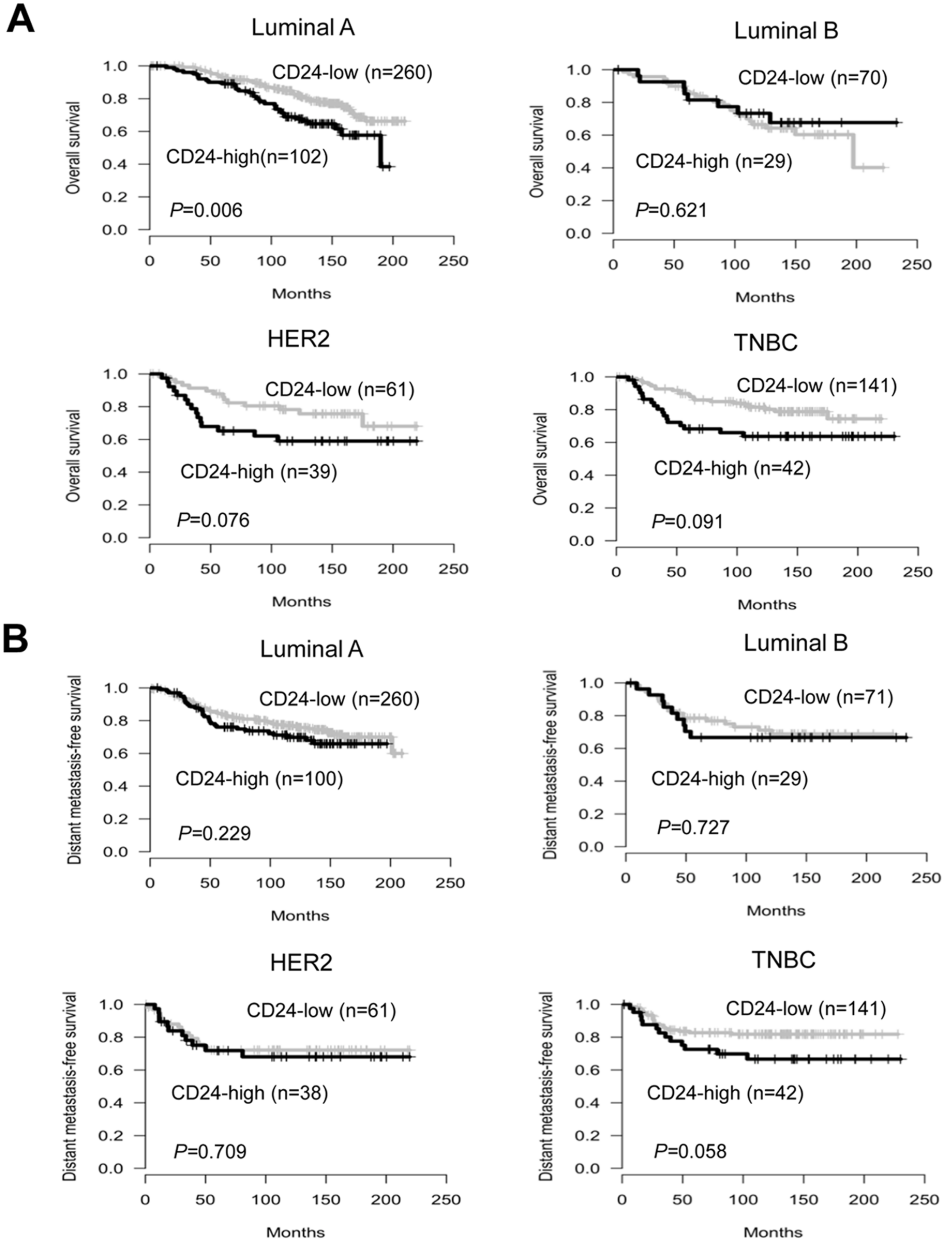
Association between CD24 expression and patient survival for different subtypes of breast cancer. Impact of CD24 expression on OS (A) and DMFS according to molecular subtypes.

We further performed subgroup analyses according to molecular subtypes of breast cancer. In the luminal A subtype, the OS of patients with high CD24 expression was significantly shorter than that of CD24-low patients (*P* = 0.006). Patients with high CD24 expression also displayed a trend towards worse OS for the HER2 (*P* = 0.076) and TNBC subtypes (*P* = 0.091) (Fig 3A). The association of CD24 expression with patient survival in the first 5, 10, and 15 years was also assessed. As a result, the impact of CD24 expression on patient survival was revealed to be time-dependent in subtypes of breast cancer. That is, the significance for OS observed in the first 5 (*P* = 0.015) or 10 years (*P* = 0.031) diminished with time for the HER2 subtype, whereas it increased with time for the luminal A subtype (S2 Fig). Marginal significance of CD24 expression with DFS was observed for the HER2 (*P* = 0.095) and TNBC subtypes (*P* = 0.087) (S3 Fig). Furthermore, in the TNBC subtype, patients with high CD24 expression had shorter DMFS than those with low CD24 expression (*P* = 0.058) (Fig 3B). Taken together, these results illustrated that the effect of CD24 expression on clinical outcome differs according to molecular subtypes, and CD24 may have a time-dependent effect on patient survival in different subtypes of breast cancer.

### Univariate analyses of DFS, OS, and DMFS in patients with breast cancer

The factors that significantly predicted poor patient survival based on univariate analysis were high CD24 expression, larger tumour size, the presence of LN metastasis, advanced pathologic stage, and higher histologic grade, but not ER or PR hormone receptor status (Table 2). High CD24 expression was significantly associated with poor DFS (HR = 1.46, 95% confidence interval [CI] = 1.10–1.94, *P* = 0.009), OS (HR = 1.57, 95% CI = 1.18–2.10, *P* = 0.002), and DMFS (HR = 1.36, 95% CI = 1.00–1.84, *P* = 0.047). A significant association of the presence of hormone receptors (ER or PR) with better clinical outcomes was found in the first 5 and 10 years but not at 15 years (data not shown). Regarding DFS and DMFS, clinical variables such as nuclear grade, chemotherapy, and radiotherapy were also significant prognostic factors, but this was not observed for OS. HER2 status was significantly associated with DFS (HR = 1.37, 95% CI = 1.03–1.84, *P* = 0.033) and marginally associated with OS (HR = 1.34, 95% CI = 0.99–1.81, *P* = 0.058).

**Table 2:** Univariate analysis of disease-free survival, overall survival, and distant metastasis-free survival in patients with breast cancer.

	Disease-free survival				Overall survival				Distant metastasis-free survival			
Variable	HR	(95% CI)		*P* value	HR	(95% CI)		*P* value	HR	(95% CI)		*P* value
Age												
<50	1.00				1.00				1.00			
≥50	0.72	0.54	0.96	**0.024**	1.08	0.81	1.43	0.599	0.75	0.55	1.01	0.058
Tumour size												
0–2	1.00				1.00				1.00			
2.1–5	1.63	1.21	2.20	**0.001**	1.63	1.19	2.24	**0.002**	1.71	1.24	2.36	**0.001**
≥5.1	2.00	1.17	3.42	**0.011**	2.60	1.58	4.28	**<0.001**	2.34	1.36	4.03	**0.002**
LN metastasis												
Negative	1.00				1.00				1.00			
Positive	2.60	1.95	3.46	**<0.001**	2.10	1.57	2.80	**<0.001**	2.94	2.16	4.01	**<0.001**
Pn												
0	1.00				1.00				1.00			
1	1.78	1.25	2.52	**0.001**	1.40	0.97	2.03	0.071	1.92	1.31	2.81	**0.001**
2	2.92	1.96	4.33	**<0.001**	2.17	1.43	3.29	**<0.001**	3.39	2.24	5.14	**<0.001**
3	4.95	3.41	7.18	**<0.001**	4.03	2.78	5.84	**<0.001**	5.77	3.90	8.55	**<0.001**
Pathologic stage												
I	1.00				1.00				1.00			
II	1.88	1.24	2.87	**0.003**	1.77	1.15	2.72	**0.010**	2.19	1.36	3.54	**0.001**
III	4.41	2.88	6.76	**<0.001**	3.91	2.53	6.04	**<0.001**	5.60	3.47	9.05	**<0.001**
Histologic grade												
1	1.00				1.00				1.00			
2	1.77	0.98	3.20	0.059	1.72	0.93	3.18	0.087	1.86	0.95	3.65	0.069
3	2.35	1.32	4.19	**0.004**	2.44	1.34	4.44	**0.003**	2.85	1.49	5.47	**0.002**
Nuclear grade												
1	1.00				1.00				1.00			
2	1.56	0.88	2.79	0.130	1.19	0.69	2.06	0.529	1.44	0.79	2.64	0.233
3	2.06	1.15	3.68	**0.015**	1.48	0.85	2.57	0.164	2.00	1.09	3.66	**0.026**
Chemotherapy												
No	1.00				1.00				1.00			
Yes	1.88	1.10	3.24	**0.022**	0.75	0.50	1.12	0.163	2.46	1.30	4.65	**0.006**
Radiotherapy												
No	1.00				1.00				1.00			
Yes	1.73	1.28	2.34	**<0.001**	1.33	0.99	1.80	0.062	1.88	1.36	2.62	**<0.001**
Hormone therapy												
No	1.00				1.00				1.00			
Yes	1.12	0.84	1.49	0.440	0.90	0.68	1.21	0.498	1.09	0.80	1.47	0.589
EIC												
Negative	1.00				1.00				1.00			
Positive	0.71	0.49	1.02	0.065	0.69	0.47	1.02	0.062	0.68	0.46	1.00	0.053
p53												
Negative	1.00				1.00				1.00			
Positive	1.00	0.74	1.34	0.981	0.87	0.64	1.18	0.373	1.05	0.77	1.44	0.749
ER												
Negative	1.00				1.00				1.00			
Positive	1.12	0.84	1.48	0.436	0.90	0.68	1.20	0.484	1.09	0.81	1.47	0.559
PR												
Negative	1.00				1.00				1.00			
Positive	1.05	0.80	1.38	0.714	0.89	0.67	1.17	0.397	0.93	0.70	1.24	0.614
HER2												
Negative	1.00				1.00				1.00			
Positive	1.37	1.03	1.84	**0.033**	1.34	0.99	1.81	0.058	1.26	0.92	1.72	0.149
Subtype												
Luminal A	1.00				1.00				1.00			
Luminal B	1.26	0.86	1.85	0.242	1.39	0.93	2.09	0.106	1.18	0.78	1.78	0.447
HER2	1.27	0.85	1.88	0.238	1.31	0.86	2.00	0.203	1.19	0.78	1.83	0.416
TNBC	0.76	0.52	1.09	0.134	1.09	0.76	1.56	0.637	0.82	0.56	1.19	0.292
CD24												
Low	1.00				1.00				1.00			
High	1.46	1.10	1.94	**0.009**	1.57	1.18	2.10	**0.002**	1.36	1.00	1.84	**0.047**

Abbreviations: LN, lymph node; EIC, extensive intraductal component; ER, oestrogen receptor; PR, progesterone receptor; HER2, human epidermal growth factor receptor 2; TNBC, triple-negative breast cancer; HR, hazard ratio; CI, confidence interval. HRs with *P* values of less than 0.05 are marked in bold.

### Multivariate analyses of DFS, OS, and DMFS in patients with breast cancer

Multivariate survival analysis was performed using Cox proportional hazard models on some factors that were found to be significant in the univariate analysis. Importantly, CD24 expression retained its statistical significance for OS (HR = 1.39, 95% CI = 1.04–1.87, *P* = 0.026) and marginal significance for DFS (HR = 1.28, 95% CI = 0.96–1.71, *P* = 0.090) at the multivariate level, indicating the CD24 overexpression is an independent negative prognostic factor in breast cancer (Table 3). Other significant prognostic factors for OS included tumour size, LN metastasis, and chemotherapy. Concerning DFS, clinical variables such as tumour size and LN metastasis were found to be independent prognostic factors with statistical significance. Notably, multivariate analysis according to subtypes illustrated that CD24 overexpression is an independent unfavourable prognostic factor for the luminal A (HR = 1.56, 95% CI = 1.00–2.41, *P* = 0.047 for OS) and TNBC subtypes (HR = 1.98, 95% CI = 1.02–3.85, *P* = 0.044 for OS; HR = 2.05, 95% CI = 1.02–4.14, *P* = 0.045 for DFS; HR = 2.18, 95% CI = 1.07–4.45, *P* = 0.032 for DMFS) (Table 3), but not the luminal B and HER2 subtypes.

**Table 3:** Multivariate analysis of disease-free survival, overall survival, and distant metastasis-free survival in patients with breast cancer.

	Disease-free survival				Overall survival				Distant metastasis-free survival			
Variable	HR	(95% CI)		*P* value	HR	(95% CI)		*P* value	HR	(95% CI)		*P* value
Age												
<50	1				1				1			
≥50	0.77	0.57	1.05	0.095	0.98	0.72	1.33	0.881	0.87	0.63	1.19	0.374
Tumour size												
0–2	1				1				1			
2.1–5	1.39	1.03	1.89	**0.032**	1.51	1.1	2.09	**0.012**	1.42	1.03	1.97	**0.035**
≥5.1	1.43	0.83	2.45	0.2	2.15	1.29	3.59	**0.003**	1.61	0.93	2.79	0.092
LN metastasis												
Negative	1				1				1			
Positive	2.29	1.7	3.09	**<0.001**	2.08	1.52	2.83	**<0.001**	2.55	1.85	3.52	**<0.001**
Chemotherapy												
No	1				1				1			
Yes	1.09	0.61	1.95	0.767	0.47	0.29	0.73	**0.001**	1.49	0.76	2.91	0.241
HER2												
Negative	1				1				1			
Positive	1.33	0.99	1.78	0.058	1.28	0.94	1.75	0.114	1.2	0.87	1.64	0.265
CD24												
Low	1				1				1			
High	1.28	0.96	1.71	0.09	1.39	1.04	1.87	**0.026**	1.17	0.86	1.6	0.31
**Luminal A subtype**												
Age												
<50	1				1				1			
≥50	1.04	0.68	1.58	0.852	1.43	0.91	2.26	0.121	1.12	0.72	1.73	0.619
Tumour size												
0–2	1				1				1			
2.1–5	1.16	0.76	1.77	0.499	1.06	0.66	1.69	0.811	1.08	0.69	1.69	0.747
≥5.1	1.4	0.63	3.07	0.407	1.5	0.64	3.53	0.352	1.48	0.67	3.29	0.337
LN metastasis												
Negative	1				1				1			
Positive	2.3	1.48	3.58	**<0.001**	2.54	1.55	4.16	**<0.001**	2.55	1.59	4.1	**<0.001**
Chemotherapy												
No	1				1				1			
Yes	1.78	0.78	4.06	0.172	0.72	0.37	1.37	0.315	2.95	1.03	8.48	**0.044**
CD24												
Low	1				1				1			
High	1.17	0.77	1.78	0.467	1.56	1	2.41	**0.047**	1.1	0.7	1.73	0.665
**TNBC subtype**												
Age												
<50	1				1				1			
≥50	0.6	0.27	1.31	0.199	0.96	0.49	1.89	0.909	0.63	0.29	1.39	0.253
Tumour size												
0–2	1				1				1			
2.1–5	2.04	0.94	4.42	0.071	2.64	1.2	5.83	**0.016**	2.15	0.95	4.83	0.065
≥5.1	4.04	1.26	12.91	**0.019**	7.51	2.62	21.54	**<0.001**	4.32	1.32	14.12	**0.016**
LN metastasis												
Negative	1				1				1			
Positive	2.3	1.19	4.46	**0.014**	2.19	1.18	4.08	**0.013**	2.65	1.33	5.26	**0.006**
Chemotherapy												
No	1				1				1			
Yes	0.61	0.17	2.17	0.441	0.61	0.2	1.88	0.386	0.58	0.16	2.1	0.409
CD24												
Low	1				1				1			
High	2.05	1.02	4.14	**0.045**	1.98	1.02	3.85	**0.044**	2.18	1.07	4.45	**0.032**

Abbreviations: LN, lymph node; HER2, human epidermal growth factor receptor 2; TNBC, triple-negative breast cancer; HR, hazard ratio; CI, confidence interval. HRs with *P* values of less than 0.05 are marked in bold.

### Epigenetic regulation of CD24 gene expression

**Fig 4:**
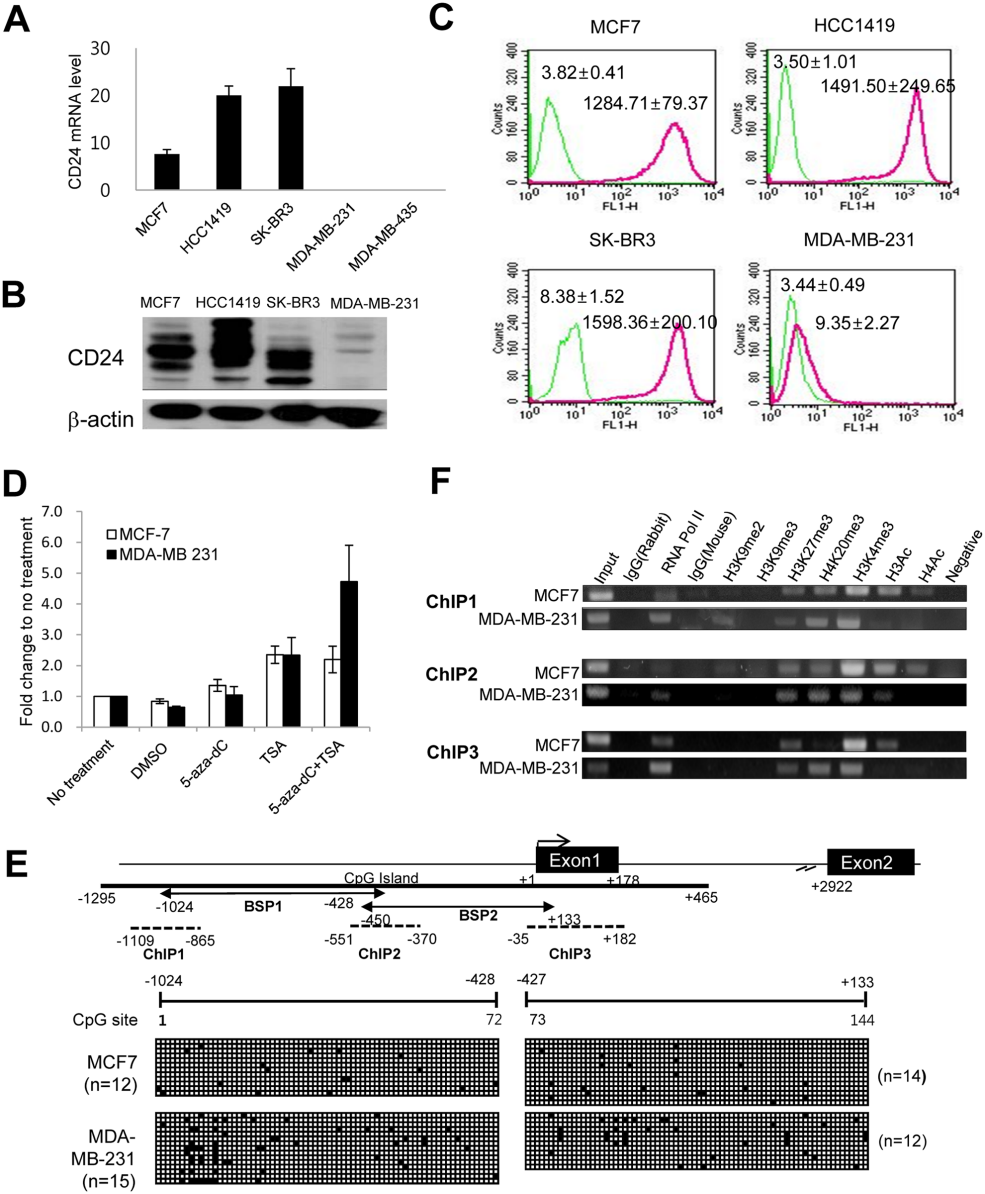
Association of epigenetic mechanism in the regulation of CD24 gene expression in breast cancer cells. Comparison of CD24 expression in breast cancer cell lines at transcript level (A), and protein level by western blotting (B) and FACS (C). (D) Effects of 5-aza-dC and TSA treatment on CD24 gene expression. (E) DNA methylation of CpG sites in CD24 promoter region. Transcription start site is indicated at the beginning of exon 1 (+1). 5′ upstream of transcription start site, exon and intron of CD24 are indicated based on the human CD24 transcript information (CD24-201, transcript ID: ENST00000606017) at Ensembl website (http://www.ensembl.org/). (F) Histone modification in CD24 promoter region in three distinct regions (ChIP 1 to ChIP 3).

Based on the association of CD24 overexpression with clinicopathological features and patient survival in breast cancer, the mechanism underlying CD24 overexpression was investigated using breast cancer cell lines. First, we determined CD24 expression at the transcript level in five breast cancer cell lines representing the subtypes of breast cancer (MCF7 for the luminal A subtype, HCC1419 for the luminal B subtype, SK-BR3 for the HER2 subtype, and MDA-MB–231 and MDA-MB–435 for the TNBC subtype) (Fig 4A). CD24 expression was also analysed at the protein level in four cell lines, and we found that CD24 protein expression was well correlated with mRNA levels and it is expressed in the cell surface membrane (Fig 4B and 4C). In line with CD24 expression using immunohistochemistry according to the subtypes of breast cancer, CD24 is highly expressed in luminal and HER2 subtypes of breast cancer cells, whereas low cell membrane expression was detected in MDA-MD–231 cells.

To determine the transcriptional regulation of CD24 expression in breast cancer cells, CD24 high-expressing MCF7 cells and CD24 low-expressing MDA-MB–231 cells were treated with epigenetic drugs, namely a DNA methyltransferase (DNMT) inhibitor (5-aza-dC) and a histone deacetylase (HDAC) inhibitor (TSA). CD24 transcript levels were significantly increased by TSA treatment alone (2.35-fold change for MCF7, 2.33-fold change for MDA-MB–231) compared to no treatment, whereas little change was induced by DNMT inhibitor treatment alone (Fig 4D). Based on the changes of CD24 expression induced by epigenetic drugs, DNA methylation and histone modification status were assessed by bisulfite sequencing and ChIP. DNA methylation status was analysed for a total of 144 CpG sites across 1,157 bp in the promoter region of CD24. The promoter region of CD24 was nearly unmethylated in both breast cancer cell lines, indicating no difference between the two cell lines (Fig 4E). Histone modification analyses in two cell lines revealed that histone acetylation (H3Ac and H4Ac) was enriched in the promoter region of MCF7 cells, whereas it was not enriched in the promoter region of MDA-MB–231 cells, demonstrating that histone acetylation is associated with CD24 expression (Fig 4F). This is also in line with the increase of CD24 expression in breast cancer cells induced by the HDAC inhibitor TSA. Interestingly, the two cell lines with high and low CD24 expression had bivalent modifications, namely the coexistence of active H3K4me3 and repressive H3K27me3 marks, which are generally found in the promoter region of stem cell-associated genes. To determine whether the difference in CD24 mRNA levels between the two cell lines is associated with the presence or absence of transcription factors, we examined luciferase reporter activity following deletion of the CD24 promoter in the two cell lines and found that luciferase activity of the various CD24 promoter fragments was similar between the two cell lines (data not shown).

## Discussion

Aberrant overexpression of CD24 during carcinogenesis and its prognostic significance in multiple types of solid tumours are well known, but the prognostic significance of CD24 expression in different subtypes of breast cancer is unclear. In the present study, we assessed the prognostic impact of CD24 expression in subgroups of breast cancer according to LN status or molecular subtypes to identify patients who can benefit from a therapy targeting CD24 and also investigated the regulatory mechanism of CD24 expression in breast cancer cells.

Our study revealed that high CD24 expression is correlated with the presence of LN metastasis and advanced pathological stage, suggesting that CD24 is associated with aggressive breast cancer. Notably, high CD24 expression was more frequently found in HER2-positive breast cancer than in HER2-negative tumours. These findings are consistent with recent studies illustrating that HER2-positive breast cancer has a predominantly CD24-high status [27] and that CD24 expression is associated with HER2 expression [28]. The low percentage of CD24-high tumours in the TNBC subtype also agrees with the enrichment of CD44^+^/CD24^−/low^ cells in triple-negative invasive breast cancer [29].

Consistent with previous studies [18, 19], we found a significant association of high CD24 expression with worse clinical outcomes in the entire group of patients with breast cancer. However, subgroup analyses demonstrated that the significance is restricted to early-stage breast cancer (pN0/pN1 or stage I). Furthermore, our study is the first to report that high CD24 protein expression is significantly associated with shorter OS in the luminal A subtype, whereas a trend towards worse outcomes was observed in the HER2 and TNBC subtypes. It is notable that the influence of CD24 expression on patient survival in the subtypes of breast cancer was time-dependent. In the HER2 subtype, the unfavourable impact of CD24 expression was restricted to the first 10 years, after which there was a reduction in the association with survival; that is, its significance diminished with time. Moreover, the trend towards worse DMFS for patients with CD24-high breast cancer of the TNBC subtype indicated the association of CD24 with distant recurrence, suggesting the possible role of CD24 in promoting metastasis in the TNBC subtype.

The association of CD24 expression with patient survival for different subtypes of breast cancer in our study suggests that targeting CD24 may confer a clinical benefit in patients with the HER2 or TNBC subtype. HER2-positive breast cancer is currently treated using the combination of HER2-targeted agents such as trastuzumab, a humanised monoclonal antibody to HER2, and chemotherapy, which prolonged patient survival. However, the majority of patients with metastatic breast cancer who initially respond to trastuzumab develop resistance to HER2-targeted therapy within 1 year [30]. Therefore, our results illustrate the possibility of CD24-targeted therapy as an effective modality for patients who are resistant to currently available HER2-targeted therapies. Interestingly, a recent study illustrated that CD24 expression can be induced by HER2 overexpression, and silencing of CD24 downregulated HER2 expression in breast cancer cells [28]. By contrast, HER2 silencing was found not to affect CD24 expression, suggesting the association of CD24 with resistance to HER2-targeted therapy. This study also revealed that CD24 downregulation by siRNA sensitises HER2-positive breast cancer cells to lapatinib, a HER2-targeted therapy, supporting the idea that targeting CD24 in combination with HER2-targeted therapy might enhance the efficacy of HER2-targeted agents.

New therapies for TNBC are urgently required, as patients with TNBC have a worse prognosis and a higher rate of recurrence with distant metastasis after chemotherapy, and hormone or HER2-targeted therapies are not effective against this subtype [31]. Our results illustrate an association of high CD24 expression with distant metastasis in the TNBC subtype. We found a trend towards shorter DFS and DMFS for patients with CD24-high tumours, and CD24 is a negative prognostic factor for DFS and DMFS in this subgroup. At first glance, our data seems to be in conflict with current assertions that CD44^+^/CD24^-/low^ subpopulation with tumour-initiating properties is positively correlated with distant metastasis in breast cancer [32], and this subpopulation enriched in TNBC subtype is associated with poor outcome of patients [29]. However, a recent study revealed that CD24 suppresses the immune response through interaction with Siglec protein expressed on immune cells [33], and this may suggest the possible contribution of CD24-Siglec pathway to the cancer immune escape. Given that the escape of cancer cells from immune surveillance is required for metastasis [34], it is possible that CD24 promotes metastasis through immune escape and this may explain the association of high CD24 expression with shorter DMFS in TNBC subtype.

The relationship CD24 expression with DFS or DMFS raises the possibility that targeting CD24 may inhibit local recurrence or distant metastasis in TNBC. A recent study by Salnikov *et al*. further supports the feasibility of CD24-targeted therapy in the treatment of cancer. This study illustrated that targeting CD24 with a monoclonal antibody inhibits tumour growth in xenograft models of lung and ovarian cancers via changes in tumour cell proliferation and angiogenesis [32]. In this study, anti-CD24 antibody also increased the efficacy of chemotherapy, suggesting the beneficial effect of combining anti-CD24 antibodies with chemotherapy [32]. However, assuming that TNBC is highly heterogeneous and the subtypes of TNBC may need different targets for better treatments, further investigation of the effect of CD24 expression in subtypes of TNBC will be required. Taken together, our data suggest that CD24 may be a promising therapeutic target for subtypes of breast cancer with worse prognoses such as the HER2 or TNBC subtype, and CD24-targeted therapy can potentiate the anti-cancer effects of chemotherapy or HER2-targeted therapy and may be effective in the treatment of HER2 therapy-resistant breast cancer or chemotherapy-resistant TNBC.

Lastly, our study first demonstrated that histone acetylation is associated with CD24 transcriptional regulation, whereas there was little correlation between DNA methylation and CD24 expression, indicating that CD24 is epigenetically regulated in association with histone modification in breast cancer cells. CD24 was recently localised to chromosome 6, whereas this gene was missing from previous genome assemblies. Therefore, the promoter sequence of CD24 was recently confirmed, and little is known regarding the mechanism underlying the transcriptional regulation of CD24, although some studies reported that CD24 mRNA expression is down-regulated by oestrogen [21] and Twist [22]. On the contrary, truncated glioma-associated oncogene homolog 1 up-regulated CD24 gene expression in breast cancer cells, thereby contributing to enhanced migration and invasiveness in breast cancer cells [35]. Importantly, HIF–1α was revealed to induce CD24 mRNA expression by binding to the CD24 promoter in prostate and bladder cancer cells under hypoxia, identifying CD24 as a transcriptional target of HIF–1α and a critical downstream effector of HIF–1α–mediated survival and metastasis [36]. Our methylation analyses in breast cancer cells correspond with current data indicating that the promoter region of CD24 is unmethylated in breast cancer cells regardless of mRNA expression levels, indicating that other mechanisms are involved in the transcriptional regulation of CD24 [23]. In particular, Kaipparettu *et al*. demonstrated that CD24 repression by oestrogen at the transcriptional level is dependent on HDACs, and HDAC inhibition can reverse this repression, supporting the involvement of histone acetylation in the transcriptional regulation of CD24 in accordance with our results. Based on the effect of HDAC inhibition on the increase of CD24 gene expression, HDAC inhibitors may enhance sensitivity to CD24-targeted therapy by de-repressing the cell surface antigens for antibody therapy.

In conclusion, our data demonstrated that high CD24 expression is significantly associated with poor survival in patients with early-stage breast cancer. We also found that CD24 overexpression is an independent unfavourable prognostic factor in breast cancer, especially for the luminal A and TNBC subtypes. Moreover, our study illustrated that CD24 mRNA expression is associated with histone acetylation but not DNA methylation. Our findings suggest that CD24 can be a promising target, especially in the HER2 or TNBC subtype of breast cancer, and targeting CD24 in combination with HER2-targeted therapy or chemotherapy may more effectively treat patients than HER2-targeted therapy or chemotherapy alone, further suggesting the effectiveness of CD24-targeted therapy in the treatment of patients resistant to conventional therapies.

## Supporting Information

S1 Fig Subgroup survival analysis according to the pathological stage. Kaplan-Meier curves for (A) disease-free survival (DFS), (B) overall survival (OS), and (C) distant metastasis-free survival (DMFS) based on CD24 expression in stage I, II, and III breast cancer.
(TIF)

S2 Fig Prognostic significance of CD24 expression at 5, 10, and 15 years. (A) Time-dependent effect of CD24 expression on overall expression (OS) for the luminal A and HER2 subtypes. (B) Time-dependent effect of CD24 expression on distant metastasis-free survival (DMFS) for the triple-negative breast cancer (TNBC) subtype.
(TIF)

S3 Fig Association of CD24 expression with disease-free survival (DFS) for different subtypes of breast cancer. (A) Impact of CD24 expression on DFS according to molecular subtypes including luminal A, luminal B, human epidermal growth factor receptor 2 (HER2), and triple-negative breast cancer (TNBC). (B) Time-dependent effect of CD24 expression on DFS in the HER2 and (C) TNBC subtypes.
(TIF)

S1 Table Primer and probe sequences used in this study (DOC)
