# Supplementary figures and images for: CD24 Overexpression Is Associated with Poor Prognosis in Luminal A and Triple-Negative Breast Cancer
Source: PLoS One. 2015 Oct 7;10(10):e0139112. doi: 10.1371/journal.pone.0139112 (PMC4596701; doi:10.1371/journal.pone.0139112)

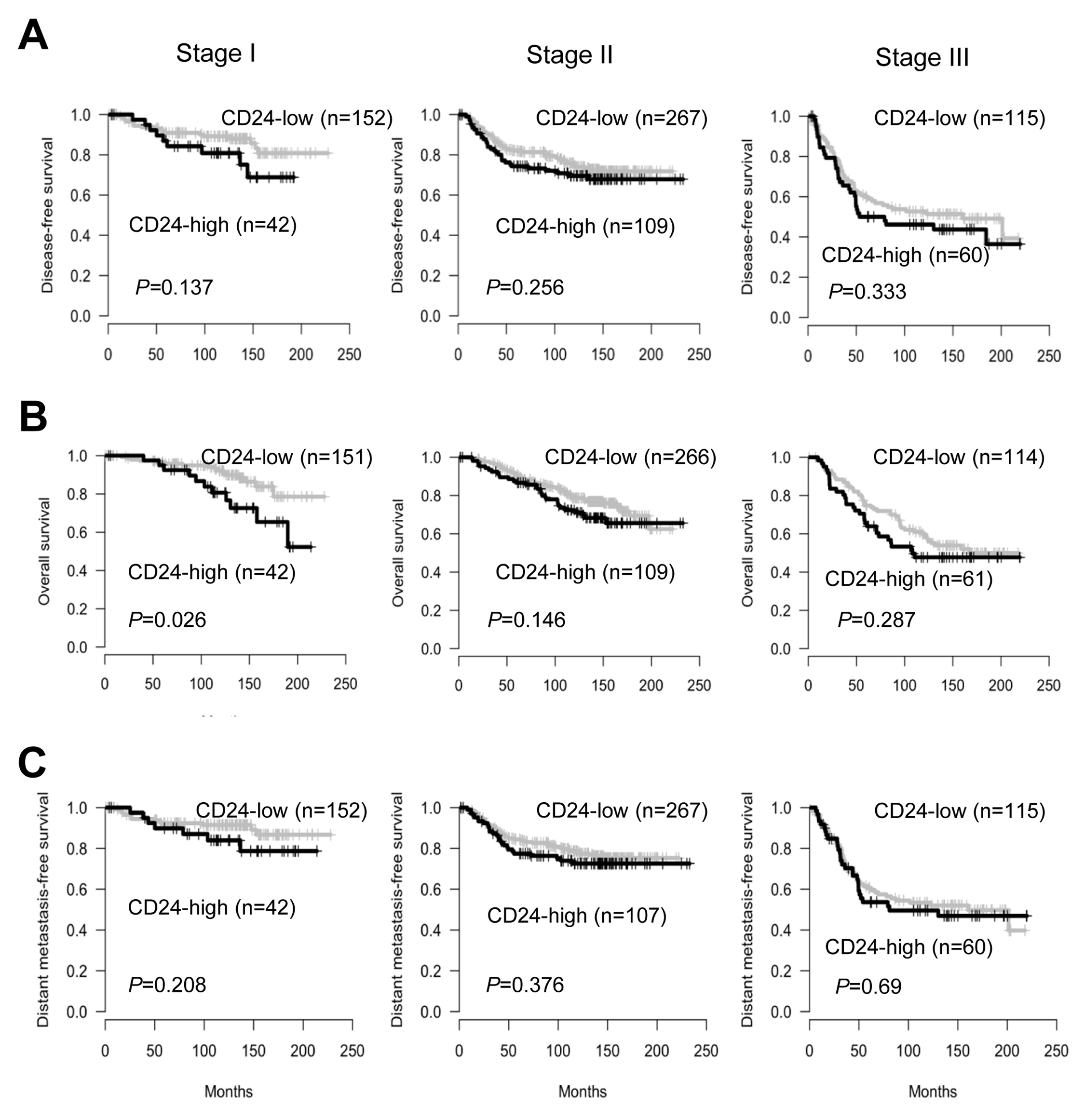

Supplement: S1 Fig — Kaplan-Meier curves for (A) disease-free survival (DFS), (B) overall survival (OS), and (C) distant metastasis-free survival (DMFS) based on CD24 expression in stage I, II, and III breast cancer. (TIF) [file pone.0139112.s001.tif]

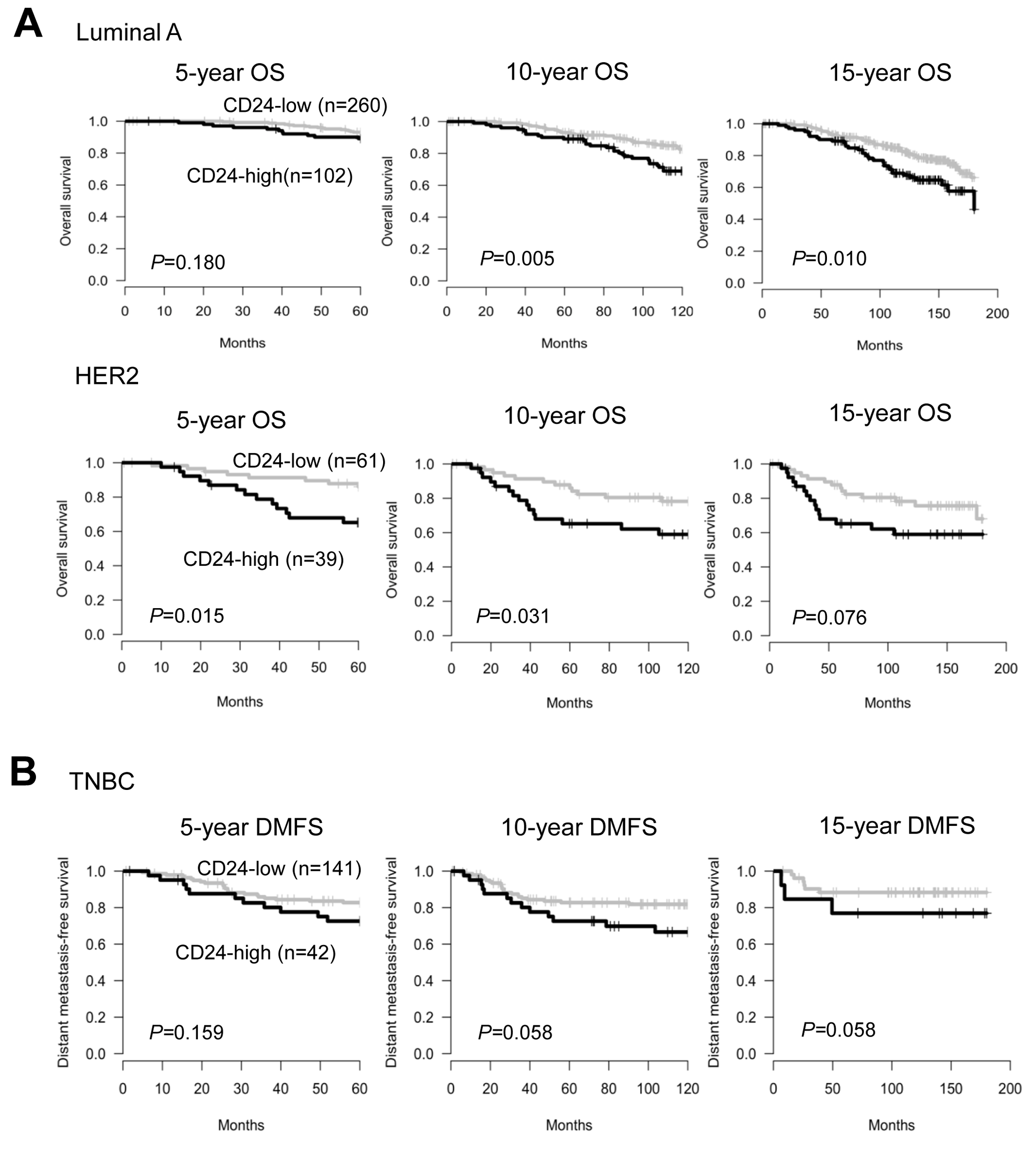

Supplement: S2 Fig — (A) Time-dependent effect of CD24 expression on overall expression (OS) for the luminal A and HER2 subtypes. (B) Time-dependent effect of CD24 expression on distant metastasis-free survival (DMFS) for the triple-negative breast cancer (TNBC) subtype. (TIF) [file pone.0139112.s002.tif]

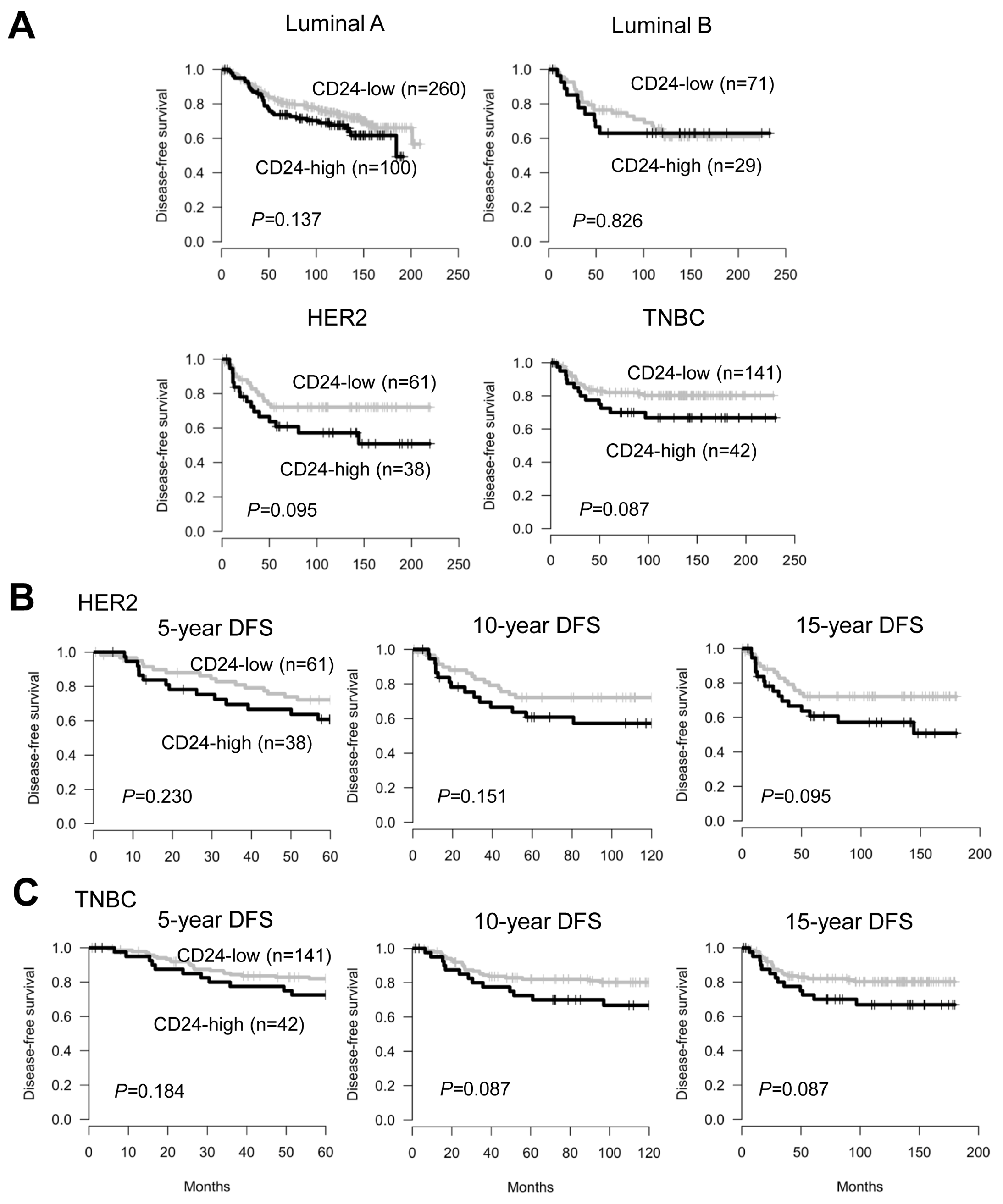

Supplement: S3 Fig — (A) Impact of CD24 expression on DFS according to molecular subtypes including luminal A, luminal B, human epidermal growth factor receptor 2 (HER2), and triple-negative breast cancer (TNBC). (B) Time-dependent effect of CD24 expression on DFS in the HER2 and (C) TNBC subtypes. (TIF) [file pone.0139112.s003.tif]
