# Supplementary material for: CD24 Overexpression Is Associated with Poor Prognosis in Luminal A and Triple-Negative Breast Cancer
Source: PLoS One. 2015 Oct 7;10(10):e0139112. doi: 10.1371/journal.pone.0139112 (PMC4596701; doi:10.1371/journal.pone.0139112)
Supplement: S1 Table — (DOC) [file pone.0139112.s004.doc]

**S1 Table.** Primer and probe sequences used in this study

| Name |  | | Sequence |  |  |  | Size (bp) | Position |
| --- | --- | --- | --- | --- | --- | --- | --- | --- |
| *CD24* |  | |  | | | |  |  |
| qRT-PCR |  | |  | | | |  |  |
| CD24 F |  | | 5-TTT GAC TAG ATG ATG AAT GCC AAT-3 | | | | 24 |  |
| CD24 R |  | | 5-GGA TGT TGC CTC TCC TTC AT-3 | | | | 20 |  |
| TaqMan probe | | | UPL #67 (FAM - CTCCAGCA-dark quencher) | | | |  |  |
| BSP | |  |  | | | |  |  |
| BSP 1 F | |  | 5-GGAGGGGAGGTTTTTGTT-3 | | | | 18 | −1024– −1007 |
| BSP 1 R | |  | 5-CCTAAAACAAATACATTACCACTCA-3 | | | | 25 | −452–−428 |
| BSP 2 F | |  | 5-AGTGGTAATGTATTTGTTTTAGG AT-3 | | | | 25 | −450–−426 |
| BSP 2 R | |  | 5-CCTAACCACCATTACTA-3 | | | | 17 | +117–+133 |
|  | |  |  | | | |  |  |
| ChIP | |  |  |  |  |  |  |  |
| ChIP 1 | |  |  |  |  |  |  |  |
| ChIP 1 F | |  | 5-CTT TAA ACG AAT GAC GGG CA-3 | | | | 20 | −1109–−1090 |
| ChIP 1 R | |  | 5-TGC AAA CTA CAG GGT TTC GG-3 | | | | 20 | −884−–865 |
| ChIP 2 | |  |  |  |  |  |  |  |
| ChIP 2 F | |  | 5-GAG AGA TAA CCC TGC CCG AG-3 | | | | 20 | −551–−532 |
| ChIP 2R | |  | 5-CCA TCT TAC CCC CGA AAA GA-3 | | | | 20 | −389–−370 |
| ChIP 3 | |  |  |  |  |  |  |  |
| ChIP 3 F | |  | 5-CAC GTC ACG GCT ATT GTG GC-3 | | |  | 20 | −35–−16 |
| ChIP 3 R | |  | 5-TCA CCT GCG TGG GTA GGA GC-3 | | | | 20 | +163–+182 |
|  | |  |  |  |  |  |  |  |
| *PAPOLA* | |  |  | | | |  |  |
| qRT-PCR | |  |  | | | |  |  |
| *PAPOLA* F | |  | 5-AAACTTTTTGAAGCTCCAAACTTCTT-3 | | | | 26 |  |
| *PAPOLA* R | |  | 5-CACCAAGCCCACCCATTC-3 | | | | 18 |  |
| TaqMan probe | |  | DYXL-AGGCGTTGTTTTTCTGTTGGTGCAC-BBQ | | | | 25 |  |

Abbreviations: qRT-PCR, quantitative reverse transcription-polymerase chain reaction; UPL, universal probe library; BSP, bisulfite sequencing PCR; ChIP, chromatin immunoprecipitation.
